# Dispositional Resilience as Mediator in Psychological Stress on Healthcare Workers: A Multi-Group Analysis of Frontline and Non-Frontline Workers

**DOI:** 10.3390/ejihpe12090089

**Published:** 2022-09-02

**Authors:** Silvia Platania, Stefania Valeria Gruttadauria, Martina Morando

**Affiliations:** Psychology Section, Department of Educational Sciences, University of Catania, 95124 Catania, Italy

**Keywords:** resilience, depression, healthcare workers, multigroup analysis, stress

## Abstract

The experiences of healthcare workers (HCWs) during COVID-19 have been characterized by psychological symptoms such as stress, anxiety and depression, compassion fatigue and post-traumatic stress, which are long-lasting. The general picture of HCWs, especially frontline workers, is that of a physically and psychologically exhausted group. The aim of the study was to examine the potential mediating role of dispositional resilience in the impact of depression, anxiety and stress on professional quality of life among HCWs during the pandemic period. We also investigated possible differences between the frontline and non-frontline HCWs. The study enrolled 487 participants from public Italian hospitals. The variables of stress and anxiety predicted all factors related to professional quality of life, against depression which positively predicted only burnout and secondary traumatic Stress. Resilience was an important mediator in all these paths and the multigroup analysis suggested statistically significant differences between frontline and non-frontline HCWs. Results emphasised the importance of caring and concern for frontline HCWs to improve their quality of life, satisfaction and have positive impacts on the quality of service and care provided. There is a need to reflect on the necessity to develop projects and protocols that address health emergencies in advance.

## 1. Introduction

The situation caused by the COVID-19 pandemic required healthcare workers to operate on an emergency status to ensure care and safe measures for people who had been exposed to the virus and to limit the spread of this infection [1].

As the pandemic started in Italy, the healthcare workers faced exhausting, long and stressful work shifts with heavy workloads. In addition, during the first weeks of the pandemic, there was a lack of any clear emergency response protocols, adequate support and the necessary personal protective equipment (PPE) [2,3].

In addition to these initial problems, additional psychological difficulties arose related to the fear of becoming infected and infecting relatives, and a general sense of helplessness towards patients’ losses.

As evidenced by other research from 2020 onwards, Italy was, and still is, among the most severely affected nations in terms of the overload of hospital patients and difficulties in managing shifts due to staff shortages [4,5,6]. This has led to great psychological stress and high health risks for health workers.

Furthermore, numerous studies have revealed the psychological stress the pandemic has had on healthcare workers [7,8].

The COVID-19 emergency exposed healthcare workers to multiple challenges and threats over time, causing symptoms of anxiety, exhaustion and stress, and even the development of depression, as confirmed by some findings on Chinese, Turkish and Spanish healthcare workers [9,10,11].

As reported by Chen and colleagues [12], p.103, more than 40 percent of healthcare workers reported experiencing anxiety symptoms; more than 46 percent reported depression, 32 percent insomnia and 69 percent high levels of stress.

According to Folkman and Lazarus [13], the greater the stress perceived by the subject, the greater the possibility of manifesting symptomatic states that cause a lower ability to manage the situation, experienced as excessive and lower perception of one’s own management skills.

As previous researchers investigating healthcare workers engaged in the SARS and MERS pandemics reported, acute distress levels are associated with other psychopathological symptoms, such as anxiety, depression and secondary traumatic stress [14,15]. The occurrence of post-traumatic symptoms combined with stressful situations is inclined to negatively impact on employee satisfaction and promote a decline in coping skills.

Healthcare and emergency workers are generally more at risk of developing secondary traumatic stress than primary trauma [16]. Several studies showed that front-line healthcare workers experienced higher levels of secondary traumatic stress scores than non-frontline healthcare workers [17,18].

Thus, the potential for infection and the perception of risk appear to worsen the healthcare worker’s discomfort as well as negative psychological symptoms [19].

A study by Wankowicz et al. [20] on a sample of workers engaged during the COVID-19 pandemic emphasised the higher risk experienced by workers in emergency departments compared to other units. Indeed, this study suggested that symptoms of anxiety and depression are more frequent and greater in frontline healthcare workers than in other workers in non-emergency departments.

Furthermore, longer working hours [21], higher workload and work intensity [22,23] could be considered as risk factors.

As a result of the pandemic, Italian healthcare workers faced a high workload due to the number of infected and dead victims. Moreover, the workload was coupled with a deficiency of proper and explicit intervention protocols, which led to a series of self-imposed protocols that were not centralised, creating confusion for both the staff and the patients in relation to the services.

However, the traumatic event does not always determine negative outcomes, and this depends on the protective factors and personal resources that each of us is able to use in the face of crisis situations.

In addition to the negative outcomes described above. Studies of frontline medical personnel have indicated that resilience, active coping strategies, planning, religion, seeking emotional and instrumental support, and self-distraction skills are positively associated with post-traumatic growth [24,25,26].

Psychological resilience is a quality that allows people to adapt and grow following traumatic situations characterised by high stress [27].

Several studies suggested that resilience is characterised by reduced vulnerability [28,29,30,31,32] and the ability to adapt to adversity, including work-related stressors [33].

In particular, resilience refers to the ability and the dynamic process to adaptively overcome difficulties without being affected by them [34]. Research has revealed that resilience was negatively correlated with stress [35] and resilient individuals are able to use positive emotions to lessen the effects of stress [36].

Based on what is asserted by the transactional theory of stress, some personality characteristics such as resilience, can influence the evaluation process which in turn mediates the stressor-stress response relationship.

Resilience appears to play a mediating role with certain psychological variables [37]. However, scarce literature examined the relation between resilience, burnout and psychological distress as interacting correlates.

Arrogante and Aparicio Zaldivar [38] identified that resilience mediates the relationships between emotional exhaustion and depersonalisation.

A study enrolling 194 nurses in Madrid [27] analysed the mediating role of resilience in the relationship between burnout and health. The authors found that resilience was important for improving the mental health of healthcare workers as well as for mitigating and neutralising the negative consequences of job stress.

Shi and colleagues [39], in a study conducted in China on medical students, found that stress and resilience played a big role in life satisfaction among Chinese medical students. In particular, resilience plays a mediating effect, and the authors suggest the importance of promoting intervention strategies aimed at increasing the degree of resilience for those who work within a healthcare context rather than strategies aimed only at decreasing perceived stress. Therefore, it is deemed necessary to reflect on the need to develop projects and protocols in the health sector that can deal with health emergencies in advance.

Thus, our study is aimed at examining the potential mediating role of dispositional resilience in the impact of depression, anxiety and stress on professional quality of life among Italian healthcare workers during the pandemic period.

Based on the relevant literature, it was hypothesised that:

**Hypothesis** **(H1).** *Variables such as stress, anxiety and depression had a direct effect on the professional quality of life of healthcare workers during the pandemic in the sample considered.*

**Hypothesis** **(H2).** *Dispositional resilience mediates the relationship between the previous antecedents and professional quality of life.*

**Hypothesis** **(H3).** *There are differences between healthcare workers having direct contact with COVID-19 patients and those who have not, in the relationship between these constructs.*

## 2. Method

### 2.1. Participants and Procedure

The study enrolled 487 (males = 280, 57.5%; females = 207, 42.5%) healthcare workers (physicians = 293, 60.2%; nurses = 194, 39.8%) from public hospitals in Italy. The mean age of the participants was 38.2 years (SD = 8.11).

Each of the participants answered preliminary questions in order to indicate and record: (1) whether they worked in close proximity with COVID-19 patients; (2) in which hospital department they worked; (3) whether their department had been reorganised into a COVID department.

For the second part of the study, the participants were divided into two main groups: workers who were in direct contact with coronavirus patients (*N* = 239, 49%) and workers who were not (*N* = 248, 51%). Eight departments were examined (infectious diseases, haematology, cardiology, psychiatry, pharmacy, general surgery, orthopaedics, emergency department).

The study was carried out in accordance with the Declaration of Helsinki and the protocol was authorised by the Internal Ethics Committee of the Department of Education Sciences (Psychology Section) of the University of Catania (Ierb-Edunict-2020/4); the data were collected between May 2020 and October 2021 and the relevant research procedures followed all the guidelines of the AIP (Italian Psychology Association) and its Ethics Council.

Participants joined the research voluntarily and were recruited through convenience sampling and hospitals were contacted through written correspondence; once approval was obtained from the human resources department, a link to the survey was posted on the companies’ social media groups (e.g., LinkedIn, Twitter). By clicking on the link, participants received an information sheet and an informed consent form which, only once accepted, led to the survey with instructions on how to complete it. Completing the survey required approximately 20 min.

### 2.2. Measures

#### 2.2.1. Depression Anxiety Stress Scales (DASS-21)

The Depression Anxiety Stress Scales (DASS-21) [40] detect three constructs: depression, anxiety and stress. Depression includes dysphoria, hopelessness, devaluation of life, lack of interest/involvement, anhedonia and inertia; anxiety relates to excitation of the autonomic nervous system, skeletal muscle effects, situational anxiety and subjective experience of anxious affects; stress relates to the presence of chronic non-specific arousal levels, relaxation difficulties, nervous excitation, irritability, agitation, hyper-activity, impatience; consists of 21 items on a 4-point Likert scale (0 = Did not apply to me at all, 1 = Applied to me to some degree, or some of the time, 2 = Applied to me to a considerable degree or a good part of time, 3 = Applied to me very much or most of the time).

The scale score is both second order (includes a total score) and second order (score for the three subscales) [41]. The calculation of the scores for each subscale is done with the classification in five severity intervals: normal, mild, moderate, severe and extremely severe. Severity labels are used to describe the full range of scores in the population, so “mild” for example means that the person is above the population mean but probably still below the typical severity of someone seeking help (i.e., it does not mean a slight level of disturbance). In the Lovibond and Lovibond [40] version of the DASS-21, the subscales are scored as follows: normal (0–4), mild (5–6), moderate (7–10), severe (11–13), and extremely severe (14+) for depression; normal (0–3), mild (4–5), moderate (6–7), severe (8–9), and extremely severe (10+) for anxiety; and normal (0–7), mild (8–9), moderate (10–12), severe (13–16), and extremely severe (17+) for stress.

#### 2.2.2. Professional Quality of Life Scale (ProQoL)

The ProQoL is a tool developed by Stamm [42,43] that measures the positive and negative aspects that affect the quality of professional life of those who are helping professionals. The ProQoL is composed of 30 tripartite items uniformly along the three dimensions identified and independent of each other: compassion satisfaction, secondary traumatic stress and burnout. The answers to the items take place via a 6-point Likert scale (where 0 indicates “never” and 5 “very often”), the participants respond to the statements referring to the last 30 days experienced in the context of their work. The score is obtained by adding the values attributed to the various items belonging to the three dimensions after having made them unidirectional.

The subscales are scored as follows: low (22 or less), moderate (23–41), high (42 or more) for compassion satisfaction scale; low (22 or less), moderate (23–41), high (42 or more) for burnout; and low (22 or less), moderate (23–41), high (42 or more), for secondary traumatic stress.

#### 2.2.3. Dispositional Resilience Scale

The DRS [44,45] Italian validation by Picardi et al. [46], is a self-completed questionnaire consisting of 15 items, scored on a 4-point scale ranging from 0 (not at all true) to 3 (completely true). The instrument includes positively and negatively keyed items covering the three conceptually important Hardiness facets of commitment, control and challenge. In addition to a total score, the DRS yields scores for three subscales: Commitment, Control, and Challenge [46] (p. 233). Scores for each subcomponent range from 0 to 15. The composite hardiness score ranges from 0 to 45.

### 2.3. Data Analysis

SPSS (version 27.0 for Windows; IBM Corp., Armonk, NY, USA) was used for the descriptive and correlations analysis of the variables in this study. Following that, we measured discriminant validity and reliability [47]. To test the reliability of the scales, we calculated the extracted mean variance (AVE) and construction reliability (CR). Acceptable values are obtained with the AVE > 0.50 [48] and the CR > 0.60. In order to test and verify the differences between the groups and the discriminant validity of the scales between unconstrained (baseline) and constrained models, we also performed a chi-squared differences test.

In order to test the indirect relationship of the dispositional resilience, mediation analysis was performed by structural equation model using AMOS 26.0 [49]. In order to verify our model, we tested two different regression models at the same time, in this way we hypothesized that the total effect of the dependent variable on the independent variable is different from the direct effect of the variable. The mediating effect of the variable was tested using a bootstrap estimation approach on 2000 samples and a percentile method corrected for 95% bias [50]. Furthermore, to estimate the moderating effect of the two different groups we applied invariance tests and multigroup analysis.

To test this multigroup analysis, it consisted of two groups extracted from the original sample: frontline and non-frontline HCWs. Afterward, the maximum likelihood (ML) estimation method was used to adjust the model individually to each group eliminating the items that did not contribute to the adjustment quality and then tested for model estimation across group). Data analysis involved three different steps: testing measurement model invariance; testing structural model invariance; and testing structural path coefficients differences.

## 3. Results

### 3.1. Descriptive Statistic, Correlation, and Reliability

The results in Table 1 showed descriptive statistics and correlation matrix for the study variables. In particular, there was a high correlation between depression and secondary traumatic stress (r = 0.41, *p* < 0.001), between burnout and secondary traumatic stress (r = 0.49, *p* < 0.001), and between stress and secondary traumatic stress (r = 0.59, *p* < 0.001). Furthermore, it is interesting to note the correlation that resilience had with the other variables in our model; in fact, there was a high negative correlation between dispositional resilience and burnout (r = −0.61, *p* < 0.001), between resilience and depression (r = −0.14, *p* < 0.001), between resilience and anxiety (r = −0.18, *p* < 0.001), and between resilience and secondary traumatic stress (r = −0.22, *p* < 0.001). Moreover, there was a positive and significant correlation between resilience and compassion satisfaction (r = 0.25, *p* < 0.001).

### 3.2. CFA to Test the Model

To determine the best-fit factor model for the data, a series of confirmatory factor analyses (CFA) were performed. Harman’s single-factor test was used to examine the common method variance (CMV) problem [51]. The first model gave a better fit for the data across all CFA fit measures. Maximum likelihood estimation was used for CFA analysis to test the structure of the constructs. We compared our model with psychological stress as a three factors antecedent (depression, stress and anxiety), dispositional resilience and with three outcome variables of professional quality of life (compassion satisfaction, secondary traumatic stress and burnout) with a model with one factor (with all items loading on a unique factor). The results revealed that the first model included six factors provided a good fit to the data: χ^2^ [305, *N* = 487] = 501.347, *p* < 0.001, χ^2^/df = 1.64, RMSEA = 0.06 (C.I. = 0.059–0.068), CFI = 0.93, GFI = 0.93, SRMR = 0.04. Moreover, the AIC and BIC values were 107.258 and 153.643.

The second CFA model considered all scales with a single-factor structure, in which all indicators loaded onto a single factor. The results of this model revealed a worse fit to the data (χ^2^ [315, *N* = 487] = 682.985, *p* = 0.018, χ^2^/df = 2.17, RMSEA = 0.91 (CI = 0.083–0.121), CFI = 0.87, GFI = 0.86, SRMR = 0.06, AIC = 401.657; BIC = 512.857).

The second model we tested does not fit our sample as well as the first, this is evidenced by both the fit indices and the difference test χ^2^ (∆χ^2^ (10) = 181.64, *p* < 0.001). The results of the difference test show that no evidence of common method bias can be observed.

### 3.3. Structural Model

To perform the mediation analysis, we used AMOS software, version 26.0 [49]. To perform this analysis tested by the CFA, we followed James, Mulaik, and Brett’s [52] recommendations and Shrout and Bolger’s [53] logic with regard to expected proximal and distal effects. The indirect effect was tested using a bootstrap estimation approach with 2000 samples and 95% bias-corrected percentile method [50].

### 3.4. Directed Effect

**Figure 1:**
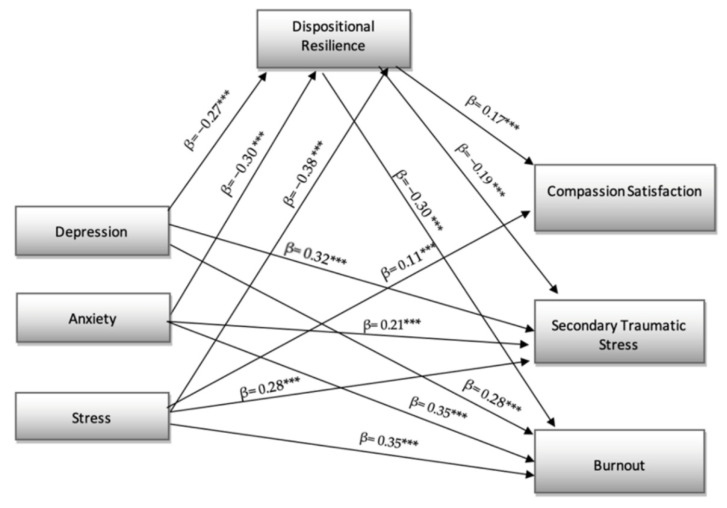
Structural model *** *p* < 0.001; non-significant links have not been reported.

The results in Figure 1 showed that there is a positive direct effect of depression on secondary traumatic stress (β = 0.32; *p* < 0.001) and burnout (β = 0.28; *p* < 0.001) and negative on dispositional resilience (β = −0.27; *p* < 0.001).

Moreover, anxiety had a positive direct effect on secondary traumatic stress (β = 0.21; *p* < 0.001) and burnout (β = 0.35; *p* < 0.001) and negative on dispositional resilience (β = −0.30; *p* < 0.001). Finally, stress had a positive direct effect on secondary traumatic stress (β = 0.28; *p* < 0.001) and burnout (β = 0.35; *p* < 0.001) and compassion satisfaction (β = 0.11; *p* < 0.001) and negative on dispositional resilience (β = −0.38; *p* < 0.001).

Furthermore, dispositional resilience had a positive direct effect on compassion satisfaction (β = 0.17; *p* < 0.001) and negative direct effect on secondary traumatic stress (β = −0.19; *p* < 0.001) and burnout (β = −0.30; *p* < 0.001). Our hypothesis 1 was confirmed.

### 3.5. The Mediation of Dispositional Resilience

To measure the indirect effects present in our sample, we performed the procedure by Hayes and Scharkow [50]. The bootstrap method is used to create 95% CIs with upper and lower levels. As shown in Table 2, the bootstrap CIs do not exceed zero.

The findings revealed that the mediating effect of resilience between predictors and outcomes is verified on all variables. There was a total mediating effect on the relationship between depression and compassion satisfaction (β = 0.04, *p* < 0.01, 95% CI [0.158, 0.414]), while partial on all other relationships. Hypothesis 2 was confirmed.

### 3.6. Multigroup Analysis

To test the moderator effect, two groups are considered: workers who have direct contact with coronavirus patients and workers who do not. To verify these group differences, it is worth investigating whether these differences result from structural differences in path coefficients across the group. It is important to check whether the measurement parameters work the same for both groups (measurement invariance test) [54,55]. Multigroup analysis was used to assess the invariance of the measurement between workers who were in direct contact with coronavirus patients and workers who were not, using chi-square difference tests for a series of nested models.

The invariance of the measurement model between the two multigroup was verified by comparing the unconstrained model (i.e., with all free parameters) with the model with constrained measurement weights (i.e., the measurement model itself). The results indicated that our model had adequate indices of adaptation in the multigroup (Table 3).

The test reveals a good fit of the model for both observed groups, although it is still possible to observe that the increase in chi-squared in the structural model indicates that there is a moderating effect of the variable defining the groups [∆χ^2^ (∆df = 28) = 40.31, *p* < 0.005] and that the structural relationships between the three latent factors would vary according to the risk situation perceived by the workers [55].

At a later stage after confirming our measurement model, we have separate structural models for each group to check if there were substantial differences in their structural relationships [54]. The results are shown in Table 4. Regarding the fit indices, both models showed similar values.

**Figure 2:**
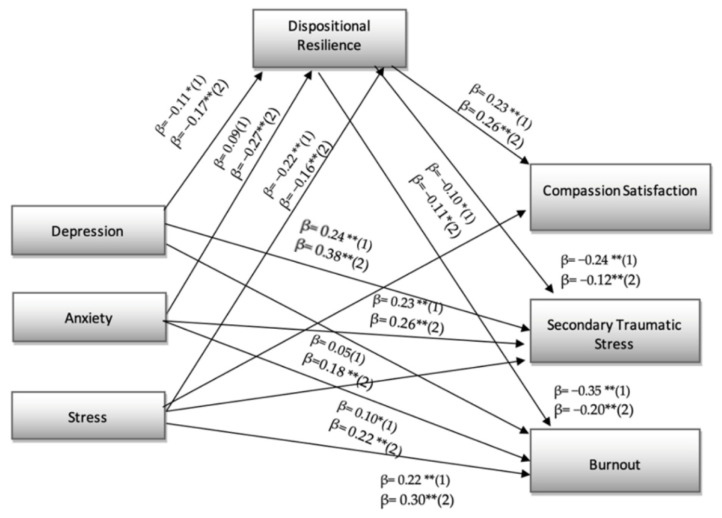
(1) Workers who were in direct contact with coronavirus patients (*N* = 239); (2) workers who were not (*N* = 248); * *p* < 0.05. ** *p* < 0.01.

Figure 2 showed that the structural relationships between the overall models are consistent between the two groups. This was possible since the regression model was plotted at two levels of the moderation variable that was split (one standard deviation above and one standard deviation below the mean). The results suggest that there are statistically significant differences between the two groups in the individual pathways. For example, the effect of depression on resilience and burnout is stronger in the group of those working in higher risk situations. In addition, resilience has a greater impact on compassion satisfaction in the less risky situations, whereas it has a smaller impact on compassion fatigue and burnout in the higher-risk contexts.

## 4. Discussion

The study is aimed at examining the potential mediating role of dispositional resilience in the impact of depression, anxiety and stress on professional quality of life among Italian healthcare workers during the pandemic period. Different studies [32,42,56] indeed showed that healthcare workers, and in particular frontline healthcare workers, are more exposed to burnout, psycho-physical disorders (insomnia, fatigue, irritability) and cognitive disorders (difficulty in concentrating) than other professions and healthcare workers in other units [20]. Potential protective factors, on the other hand, were compassion satisfaction, resilience and psychological capital, indicating that high levels of these constructs are capable of keeping healthcare workers strong and stable. Given these interesting reflections, it seemed appropriate to question the health and quality of professional life of Italian healthcare workers, also investigating possible differences between the frontline and non-frontline healthcare workers.

Emergency situations, as pandemic situations, usually tend to have an immediate effect on healthcare workers, resulting in psychological distress. Findings from the present study revealed, in fact, that the sample of healthcare workers examined presented clinically relevant symptoms of depression, which can be classified in a category of medium severity. These findings are in line with others carried out in other contexts, in which participants tested positive for depression during the COVID-19 pandemic [57]. Another study [57] found that 45% of a sample of nurses having close proximity with infected people suffered from depression and 14% had moderate to severe depression. From these findings it could be inferred that nurses having direct contact with infected persons suffered from fears of infection and mortality [58]. Furthermore, the contribution by Wankowicz et al. [20] emphasised the increased risk experienced by healthcare workers exposed to SARS-V-2-infected patients, of suffering from anxiety and depression more significantly than healthcare workers working in other units.

In addition to these findings, the sample examined showed moderate levels of anxiety and stress. Anxiety is one of the most common psychological effects during the COVID-19 public health crisis, and it is pervasive among healthcare providers. These results are consistent with other studies conducted nationally and internationally. A study conducted by Wang and colleagues [59] in China, with frontline healthcare workers working with COVID-19, revealed that post-traumatic stress is more likely to develop as a result of particularly threatening experiences, manifested through typical symptoms such as re-experiencing such events, being on the alert and the continuous feeling of threat [60]. An intense and critical experience, such as the COVID-19 pandemic, is thus capable of acting and impacting on post-traumatic stress levels among healthcare workers, and this level of stress affects these categories of workers more than others [60,61,62]. These findings are also found in studies with different epidemics (e.g., SARS in 2003), where 25.8% of physicians had symptoms of post-traumatic stress [63] and a diagnosis of PTSD 2 months after the outbreak [64].

In line with these results, the sample also presents moderate levels of burnout and low levels of secondary traumatic stress. Compared with other studies, the results obtained are not very different; on the contrary, they confirmed the trend and the excessive workload produced by the pandemic situation. In a study involving Portuguese health workers for example, 21.6% showed moderate burnout and 47.8% high burnout. Unsurprisingly, SARS-CoV-2 posed unprecedented challenges to healthcare workers, directly affecting their stress levels and burnout. Previous research on burnout indeed found that the highest burnout rates occur in emergency situations, affecting frontline workers the most [20]. Therefore, in the case of a pandemic, these levels appear to become moderate or high as in the case of this sample. Caution is important since high burnout levels often result in negative consequences such as reduced quality of care [65] and increased frequency of errors in healthcare [66,67].

In contrast to other studies [11,68,69,70] in the literature, the present sample exhibited no high levels of secondary traumatic stress. This result, apparently detached from the rest, could be interpreted as an increased perception of stress on a personal level. Secondary traumatic stress (STS), in fact, refers to secondary, work-related exposure to extremely stressful events. The development of problems due to exposure to other people’s trauma is quite rare, but it happens to many people who deal with those who have experienced extremely or traumatically stressful events. However, given the mandatory distancing and strong protective measures, and the massive exposure to traumatic and dangerous events, such workers are more likely to experience primary than secondary traumatic exposure. Interpreted in this sense, the results seem more consistent with those already reported.

For the last of the factors related to professional quality of life, compassion satisfaction reported moderate levels, posing for all intents and purposes as a protective factor to be enhanced. This result could be explained by interpreting healthcare as vocational work, which is why the participants may be so satisfied. However, even though there are many participants who experienced high compassion satisfaction, there could be many negative consequences at the same time. It may seem contradictory that participants reported moderate levels of compassion satisfaction and a range of psychological symptoms. However, several studies present the same results confirming a trend and a real possibility [71]. For example, a study of healthcare workers employed in critical incident services revealed that the participants, although at risk of burnout or compassion fatigue, still showed a high level of compassion satisfaction [72]. This might be because although these workers recognise the level of stress associated with their work, it provides significant rewards that somehow manage to mitigate stress and burnout.

In relation to H1, the variables stress and anxiety predict all factors related to professional quality of life (burnout, compassion satisfaction and secondary traumatic stress). In contrast, the variable depression positively predicts only burnout and secondary traumatic stress. Several studies revealed that the risk factors, or those that showed a positive relationship with burnout, are mainly anxiety and depression [73,74,75], insomnia [74,76], and moral impairment [74]. Job stress also seems to significantly influence burnout [77] just as the lack of personal protective equipment influenced emotional exhaustion [78,79]. High levels of burnout occurred in healthcare workers in conjunction and association with the increase in compassion fatigue and secondary traumatic stress, resulting from the COVID-19 pandemic. Thus, our first hypothesis was confirmed.

The literature on the issue depicted dispositional resilience as an adaptive quality possessed by humans, which enables them to thrive following the experience of traumatic and high-stress situations [32,80].

Resilience is thus an adaptive personal resource that enables a person to cope with various significant stressors, including work-related stressors [33,80]. Adapted to the workplace, resilience has been defined as the “positive psychological ability to recover, to ‘bounce back’ from adversity, uncertainty, conflict, failure or even positive change, progress and increased responsibility” (p. 702) [34]. In the specifics of the present study, the sample examined showed average values for resilience, demonstrating how this is a characteristic already present but to be improved and encouraged. According to several studies, the importance of resilience competence resides in its role in minimising and protecting against stress-related negative effects such as burnout syndrome [81,82,83]. The contribution of West et al. [84,85] with a sample of 5445 physicians, for example, suggested that resilience is inversely associated with burnout symptoms, but that at the same time burnout is also experienced among those with adequate and good levels of resilience.

The H2 test’s findings are very interesting. The findings indicate, indeed, that resilience mediates the relationship between anxiety, depression and stress and the quality of work life: in particular, it emerged a total mediating effect on the relationship between depression and compassion satisfaction, while partial on all other relationships.

Resilience is an important mediator in all these paths, but when the employee is overworked it might no longer be sufficient, especially with regard to the effect of depression on burnout. However, resilience can also effectively impact on compassion satisfaction in emergency situations by reinforcing it. These findings supported our second hypothesis.

The results from the H3 test suggested that there are statistically significant differences between the two groups considered, i.e., frontline and non-frontline healthcare workers. For example, the effect of depression on resilience and burnout is stronger in the group of those working in higher risk situations. These results are in line with other studies conducted on the topic, which emphasise that emergency situations and specific hospital units experienced higher levels of anxiety, depression and stress [20,21,22,23]. Furthermore, resilience had a greater impact on compassion satisfaction in less risky situations, while it had less impact on secondary traumatic stress and burnout in higher risk settings. This result is very interesting, as it emphasises the importance of caring and concern for frontline healthcare workers in order to improve their quality of life, satisfaction [86,87] and also have positive impacts on the quality of service and care provided [88,89,90].

## 5. Conclusions

The experiences of healthcare workers during COVID-19 are not completely unknown to society. Indeed, there is an opportunity to learn from the experiences of previous pandemics with an aim to provide better support to frontline healthcare workers.

This study, together with others carried out worldwide, showed that healthcare workers experience psychological symptoms such as stress, anxiety and depression, compassion fatigue and post-traumatic stress, and have been suffering from this effect for a considerable length of time. The overall picture of healthcare workers, especially frontline workers, is of a physically and psychologically exhausted group of workers.

This study attempts to recommend the implementation of psychological support for these workers, but above all a series of prompt interventions that could assist and support these workers.

Preventive interventions and training programmes should be developed to guide healthcare workers to better cope with traumatic events at work. Such interventions are emphasised to be important both for the wellbeing and health of healthcare workers, but also for the effective use of coping strategies and an improved quality of patient care.

Not many studies have investigated the mediating effect that resilience can have in fostering health worker resilience to traumatic effects such as pandemics. The studies that have so far examined the mediating effect of resilience, however, all agree that it is more important to develop programmes and intervention strategies aimed at stimulating the resilience of health workers rather than strategies aimed at decreasing stress. In this sense, providing prevention and care for the health worker who always has to deal with high-stress situations is of fundamental importance.

Having a psychologically healthy healthcare workforce is an advantage for them and for the whole community: it means providing more resources to society and in particular to the healthcare workforce.

Therefore, urgent action is needed to protect the physical and psychological health of healthcare workers, especially those on the frontline of the COVID-19 pandemic. This will ensure the maintenance of a robust, functional and efficient health system capable of facing challenges such as the current one and others of a similar level. This commitment is also important in view of the enormous sacrifices and commitment that healthcare workers have provided for all for years.

### Limits and Practical Implications

Although promising results emerged from the study, it is important to emphasise certain limitations. First, the cross-sectional nature of the design employed. Therefore, a longitudinal study is needed to verify the long-term effects, especially with regard to secondary traumatic stress. Second, the sample is too small and contextualised, so it would be appropriate in future studies to extend it to include other regions and differentiations. Moreover, the distinction between the groups used for the sample examined ought to relate beyond direct contact with the patient and consider other variables that could change its impact (age, gender, etc.). It is also important to mention that the impact that organisational support, in terms of the psycho-social safety climate, might have on the management of unforeseen events through which the worker perceives risk, should also be included in a more comprehensive study.

In any case, the results of this study make an important contribution to the assessment of the physical and psychological health situation of these workers and emphasise the importance of investing in and enhancing personal skills and organisational support. We must organise a better response to current and future challenges, and for this, it is necessary to open the door to further studies and research on the topic.

## Figures and Tables

**Table 1:** Descriptive statistic, correlation, and reliability (*N* = 487).

	M	SD	α	AVE	CR	1	2	3	4	5	6
1. Secondary traumatic stress	17.1	5.2	0.92	0.70	0.88	1					
2. Burnout	25.7	4.9	0.89	0.64	0.82	0.49 **	1				
3. Resilience	25.1	4.6	0.79	0.66	0.87	−0.22 **	−0.61 **	1			
4. Depression	4.3	1.3	0.82	0.60	0.81	0.41 **	0.21 **	−0.14 **	1		
5. Anxiety	3.8	1.2	0.71	0.72	0.91	0.45 **	0.14 *	−0.18 **	0.29 **	1	
6. Compassion satisfaction	29.5	8.7	0.78	0.65	0.85	−0.35 **	−0.20 **	0.25 **	−0.22 **	−0.13 *	1
7. Stress	8.2	2.3	0.85	0.63	0.82	0.59 **	0.28 **	−0.34 **	0.32 **	0.36 **	−0.37 **

** correlations are significant at the *p* < 0.001 level; * correlations are significant at the *p* < 0.05 level; α = Alpha di Cronbach; AVE = Average Variance Extracted; CR = Composite Reliability.

**Table 2:** Standardized indirect effects from psychological stressor to professional quality of life through dispositional resilience.

Predictor	Mediator	Outcome	β	SE	BC 95% CI	
					LL	UL
Depression →	Dispositional Resilience →	Compassion satisfaction	0.04 **	0.03	0.158	0.414
Depression →	Dispositional Resilience →	Secondary traumatic stress	0.06 ***	0.03	0.162	0.718
Depression →	Dispositional Resilience →	Burnout	0.04 **	0.02	0.265	0.476
Anxiety →	Dispositional Resilience →	Secondary traumatic stress	0.07 ***	0.05	0.043	0.189
Anxiety →	Dispositional Resilience →	Burnout	0.05 **	0.03	0.056	0.041
Stress →	Dispositional Resilience →	Compassion satisfaction	0.07 ***	0.05	0.125	0.369
Stress→	Dispositional Resilience →	Secondary traumatic stress	0.06 **	0.04	0.168	0.412
Stress →	Dispositional Resilience →	Burnout	0.06 ***	0.04	0.197	0.368

*** *p* < 0.001, ** *p* < 0.01.

**Table 3:** Multigroup analysis: Testing for measurement invariance across workers who were in direct contact with coronavirus patients (*N* = 239) and workers who were not (*N* = 248).

Measurement Model	χ^2^	df	∆χ^2^	∆df	NFI	CFI	RMSEA
Multigroup model for the total sample	125.84	107	-		0.90	0.96	0.051
Unconstrained model	134.91	116	9.07	9	0.90	0.96	0.049
Measurement model	145.91	128	20.07	21	0.89	0.95	0.049
Structural model	166.15	135 *	40.31 ***	28	0.88	94	0.052

*** *p* < 0.001, * *p* < 0.05. NFI = Normed fit index; CFI = Comparative fit index; RMSE = Root mean square error of approximation.

**Table 4:** Multigroup analysis: Testing for path coefficients invariance across workers who were in direct contact with coronavirus patients (*N* = 239) and workers who were not (*N* = 248).

Structural Model	χ2	df	∆χ^2^	∆df
Model 1: Baseline model	149.01	130	-	-
Model 2: Factor loadings and all path coefficients invariant	165.32	135	16.31 **	5
Model 3: Path coefficient DE→ DR unconstrained	158.31	134	7.01 **	1
Model 4: Path coefficient DE→ CS unconstrained	160.25	134	1.94	1
Model 5: Path coefficient DE→ STS unconstrained	162.11	134	1.86	1
Model 6: Path coefficient DE→ BO unconstrained	157.54	134	4.57 *	1
Model 7: Path coefficient ANX→ DR unconstrained	163.24	134	5.7 **	1
Model 8: Path coefficient ANX→ CS unconstrained	158.25	134	4.99 *	1
Model 9: Path coefficient ANX→ STS unconstrained	162.78	134	4.53 *	1
Model 10: Path coefficient Stress → DR unconstrained	165.84	134	3.06 *	1
Model 11: Path coefficient Stress → CS unconstrained	163.14	134	2.7	1
Model 12: Path coefficient Stress → STS unconstrained	165.25	134	2.11	1
Model 13: Path coefficient Stress → BO unconstrained	162.36	134	2.89	1
Model 11: Path coefficient DR → CS unconstrained	165.84	134	3.48 *	1
Model 11: Path coefficient DR→ STS unconstrained	160.25	134	5.59 **	1
Model 11: Path coefficient DR→ BO unconstrained	155.89	134	4.36 *	1

** *p* < 0.001. * *p* < 0.05.
